# Hyperglycemia exerts disruptive effects on the secretion of TGF-β_1_ and its matrix ligands, decorin and biglycan, by mesenchymal sub-populations and macrophages during bone repair

**DOI:** 10.3389/fdmed.2023.1200122

**Published:** 2023-06-26

**Authors:** Norhayati Yusop, Ryan Moseley, Rachel J. Waddington

**Affiliations:** School of Dentistry, Cardiff University, Cardiff, United Kingdom

**Keywords:** mesenchymal stromal cells, bone repair, osteoblasts, macrophages, hyperglycemia, type 2 diabetes mellitus

## Abstract

**Introduction:**

Bone has a high capacity for repair, but for patients with uncontrolled type 2 diabetes mellitus (T2DM), the associated hyperglycemia can significantly delay osteogenic processes. These patients respond poorly to fracture repair and bone grafts, leading to lengthy care plans due to arising complications. Mesenchymal stromal cells (MSCs) and M2 macrophages are both major sources of transforming growth factor-β_1_ (TGF-β_1_), a recognized mediator for osteogenesis and whose bioavailability and activities are further regulated by matrix small leucine-rich proteoglycans (SLRPs), decorin and biglycan. The aim of this study was to investigate how *in vivo* and *in vitro* hyperglycemic (HGly) environments can influence the levels of TGF-β_1_, decorin, and biglycan during bone repair, with additional consideration for how long-term glucose exposure and cell aging can also influence this process.

**Results:**

Following bone healing within a T2DM *in vivo* model, histological and immunolabeling analyses of bone tissue sections confirmed delayed healing, which was associated with significantly elevated TGF-β_1_ levels within the bone matrices of young diabetic rats, compared with their normoglycemic (Norm) and aged counterparts. Studies continued to assess *in vitro* the effects of normal (5.5 mM) and high (25 mM) glucose exposure on the osteogenic differentiation of compact bone-derived mesenchymal stromal cells (CB-MSCs) at population doubling (PD)15, characterized to contain populations of lineage-committed osteoblasts, and at PD150, where transit-amplifying cells predominate. Short-term glucose exposure increased TGF-β_1_ and decorin secretion by committed osteoblasts but had a lesser effect on transit-amplifying cells. In contrast, the long-term exposure of CB-MSCs to high glucose was associated with decreased TGF-β_1_ and increased decorin secretion. Similar assessments on macrophage populations indicated high glucose inhibited TGF-β_1_ secretion, preventing M2 formation.

**Discussion:**

Collectively, these findings highlight how hyperglycemia associated with T2DM can perturb TGF-β_1_ and decorin secretion by MSCs and macrophages, thereby potentially influencing TGF-β_1_ bioavailability and signaling during bone repair.

## Graphical Abstract

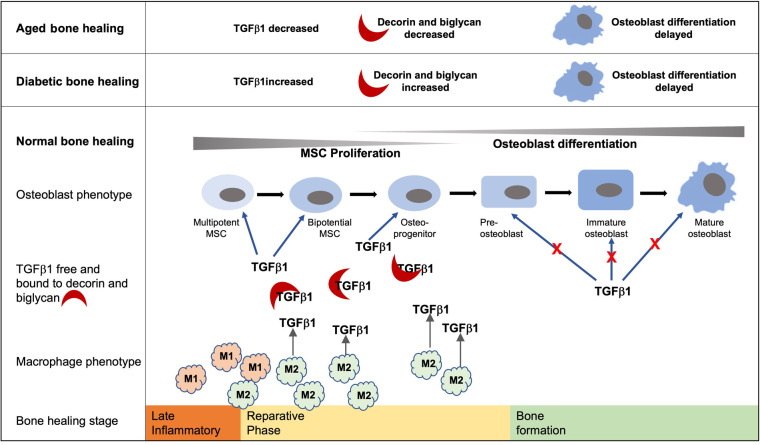

Bone healing follows an initial inflammatory phase followed by a reparative phase during which TGF-β_1_ within the healing extracellular environment promotes MSC proliferation and early commitment of MSCs to the osteoblast lineage. Conversely, TGF-β_1_ signaling inhibits later stages of osteoblast differentiation during the deposition of a bone matrix. Increased and prolonged levels of TGF-β_1_ and decorin and biglycan within the diabetic bone healing environment have the potential to delay the development of mature osteoblasts. Likewise, the decreased levels of TGF-β_1_ associated with the aged diabetic healing environment hinder the early stages of osteoblast development and hence bone formation. While they are not the only source of TGF-β_1_, hyperglycemic environments impede the development of the M2 phenotype and are known to play important roles in bone formation during the reparative stage.

## Introduction

The surgical insertion of a dental or orthopedic implant into osseous tissue leads to the activation of a biological sequence of events, where extracellular matrix (ECM) components play central regulatory roles (1, 2). Following a classical bone repair response, associated trauma to the bone initiates the activation of the complement cascade and the migration of platelets, leading to the formation of a fibrin clot which holds a source of growth factors that orchestrate the infiltration of immuno-inflammatory cells, such as neutrophils and macrophages (1, 2). Subsequent evolving changes in the growth factor milieu trigger the reparative stages involving the migration, proliferation, and osteogenic differentiation of mesenchymal stromal cells (MSCs), which facilitate mineralized bone matrix deposition (2–4). Critical to these latter stages in promoting successful bone healing is the transition of the M1 macrophage to the M2 phenotype, where prolonged presence of M1 macrophages potentiates extended inflammation and hence delays the reparative processes (5, 6).

Type 2 diabetes mellitus (T2DM) is recognized as a systemic disease arising out of poor glycemic control, leading to hyperglycemia and cellular insulin resistance (7, 8). Consequently, uncontrolled T2DM contraindicates the development and/or exacerbation of many bone-related pathologies, including the reparative processes of implant osseointegration (9, 10). Indeed, *in vivo* studies investigating implant osseointegration using animal models of T2DM have consistently indicated reduced bone repair capacity compared to normal animal controls (11–14). Further analysis of the osseous tissues has suggested that impaired diabetic bone healing is due to the delayed onset of osteogenic reparative responses (12). In addition, the diabetic environment is associated with a delayed but sustained increase in the cellular secretion of transforming growth factor-β_1_ (TGF-β_1_), interleukin-1β (IL-1β), and tumor necrosis factor-α (TNF-α) and prolonged presence of macrophages. Indeed, recent studies have indicated that increased M1 and decreased M2 macrophage polarization may be responsible for delayed bone healing around implants in patients with T2DM (15, 16), indicative of cells and matrix signaling factors with potential to propagate the pro-inflammatory environment associated with T2DM (5, 6, 12). T2DM is also a systemic disease that is predominantly associated with an aging population, where aging is now established to result in replicative exhaustion and a loss in regenerative potency by MSCs (17, 18), compounding the issue further.

While the mechanisms of action are not fully understood, it is generally agreed that TGF-β_1_ participates in the regulation of immune response, angiogenesis, cell migration, osteoprogenitor cell proliferation, differentiation, and cell survival and in stimulating collagen-rich, osteoid formation within bone-healing sites (19–21). Conversely, during the latter stages of repair, TGF-β_1_ is considered to be an inhibitory factor toward the further differentiation of osteoblasts associated with deposition of the mineralized matrix (22–24), although the further differentiation of osteoblasts into osteocytes is proposed to be aided by TGF-β_1_ in preventing osteoblast apoptosis (25). TGF-β_1_ bioavailability and activities are further regulated through its interactions with the localized extracellular matrix (ECM) (26–28). Studies have established that decorin and biglycan, as members of the small leucine-rich proteoglycan (SLRP) family, have the ability to bind key growth factors critical for successful bone healing, such as TGF-β_1_ (29, 30) and bone morphogenetic proteins (BMPs) (31–33). Sequestering TGF-β_1_ within biological matrices thereby regulates its release and presentation to cell surface receptors in activating TGF-β_1_ signaling pathways (26, 30). In addition, biglycan has been proposed to directly stimulate bone formation via the dual signaling mechanisms of BMP/TGF-β_1_ and canonical Wnt/β-catenin pathways (34). Meanwhile, decorin has long accepted roles in attenuating the potent fibrotic activity of TGF-β_1_ (28). Via interaction with insulin-like growth factor-1 receptor (IGF_1_R) and integrin α_2_β_1_, decorin is also proposed to support angiogenesis (35). However, more recent research has identified an additional complexity in the roles of decorin and biglycan, suggesting they can act as molecular switches to support chronic inflammation or its resolution, depending upon receptor selectivity (36), thereby promoting or antagonizing TGF-β_1_ regulation of osteo-immunological events critical for bone repair.

Several *in vitro* studies have previously demonstrated the negative impact of high glucose exposure on the repair capabilities of bone marrow mesenchymal stromal cells (BM-MSCs) derived from the perivascular niche, including reduced proliferation, colony-forming efficiency, and osteogenic differentiation, with increased apoptosis, which ultimately impair bone mineralization *in vivo* (37–40). However, cells within the endosteal and periosteal niche, lining bone surfaces also play important roles in facilitating bone repair, where sub-populations of more committed, lineage-restricted osteoprogenitor cells have been proposed to act as “first responders” during mineralized tissue repair (41–43). Recent *in vitro* characterization of heterogeneous MSC populations from the endosteal/periosteal niche of rodent compact bone (CB-MSCs) has identified that high glucose conditions exert a limited effect on proliferative and stemness characteristics, although osteogenic differentiation and mineralization were impaired (44).

Against this background, the aim of this study was to investigate how a hyperglycemic environment, associated with diabetes, can influence the bioactivity and hence potential activity of TGF-β_1_ in driving osteogenesis. In addition, this study investigated how the effects of long-term exposure to high glucose and cell aging can further influence the bioavailability of TGF-β_1_ within a hyperglycemic environment. To achieve these aims, we first utilized an *in vivo* T2DM rodent model of impaired bone repair (11, 12) to ascertain how hyperglycemic conditions influence TGF-β_1_ levels in young and aged tissues during implant osseointegration. To gain insights into whether perturbations in diabetic bone healing are mediated via changes in TGF-β_1_ bioactivity, *in vitro* studies further considered the short- and long-term effects of high glucose exposure on TGF-β_1_ expression/secretion by CB-MSCs and how decorin and biglycan could influence TGF-β_1_ bioavailability. As high glucose exposure can also dysregulate macrophage phenotype development and function (45–47), the influence of high glucose exposure on TGF-β_1_ expression/secretion in influencing the M1/M2 phenotype was also investigated.

## Materials and methods

### *In vivo* analysis of bone repair and TGF-β_1_ secretion

All tissue samples analyzed were gifted by Professor J Okazaki, representing residual tissue arising from a separate study. A total of 12 male diabetic GK rats (six aged 10 weeks and six aged 18 weeks) and 12 age-matched male Wistar rats (Shimizu Laboratory Supplies Co. Ltd., Kyoto, Japan) were used. Raised levels for blood glucose and HbA1c were confirmed in the GK rats from the age of 3 weeks (11). All animal experiments performed were reviewed and approved by the Animal Committee of Osaka Dental University (approval number 08-03009). Sterile titanium alloy (Ti–6A1–4V) implants (length 17.0 mm, diameter 1.2 mm; SNK 123 screwpost Ti-tan R, Dentsply-Sankin K.K., Tokyo, Japan) were placed into the socket of a freshly extracted lower incisor, as previously described (11, 12). At 3 and 9 weeks after implant placement, three rats from each experimental group were euthanized by an intraperitoneal injection of sodium pentobarbital 30 min after an intraperitoneal injection of the anticoagulant sodium heparin (Novo-Heparin Injection 1,000®; Mochida Pharmaceutical, Tokyo, Japan). Tissues were fixed by perfusion with 10% neutral buffered formalin (11) and the mandible tissues containing the implants dissected.

### Histology and immunogold labeling

Implants were gently unscrewed from mandibles. Using a bone saw, tissue blocks with a width of 2 mm were cut along the entire length of the mandible in an axis running perpendicular to the implant socket. Tissue pieces were demineralized with 10% formic acid for 72 h and then dehydrated by passing them through 70%–100% graded alcohol and finally into xylene, before embedding them in paraffin. Sections of 5 µm were cut and mounted on poly-L-Lysine coated glass slides (Sigma-Aldrich, Poole, UK), stained with hematoxylin and eosin (H&E), and mounted using a DPX mounting medium (ThermoFisher Scientific, Paisley, UK). Sections were examined using light microscopy.

Alternatively, tissue blocks were further cut in the sagittal and coronal planes to produce four pieces, each containing tissue that had been adjacent to the implant. Tissue blocks were demineralized using 6% ethylenediaminetetraacetic acid (EDTA) solution (pH 7.4), for 4 weeks. Specimens were washed in 0.01 M phosphate buffered saline (PBS) before dehydration through increasing concentrations of ethanol and then embedded in Lowicryl HM20 (Agar Scientific Elektron UK Ltd., Stansted, UK), using a progressive lowering of temperature (PLT) protocol. Full polymerization of the resin was achieved by using indirect ultraviolet (UV) light at 35°C for 24 h and direct UV light for another 72 h at room temperature (48).

Immunogold labeling of antigens was visualized using transmission electron microscopy (TEM), following the previously reported method of Hobot and Newman (48). Sections of 4 µm were cut from the Lowicryl embedded tissue blocks and collected onto 400 mesh nickel grids. Sections were washed with 0.01 M PBS (50 µl) for 10 min, blocked with 0.6% w/v bovine serum albumin (BSA), diluted in 0.01 M PBS (pH 7.4, 50 µl) for 10 min, and then incubated with an affinity-purified polyclonal rabbit IgG anti-rat TGF-β_1_ antibody (diluted 1:10; Santa Cruz Biotechnology, Dallas, TX, USA) for 1 h. Goat anti-rabbit IgG (Sigma-Aldrich), conjugated to 10 nm colloidal gold, GAR-10 (Insight Biotechnology, Wembley, UK), was prepared as previously described (48), diluted 1:5 in filtered 20 mM Tris-HCl buffer (pH 8.2) and centrifuged at 10,000 g for 4 min. This secondary antibody was incubated with sections for 1 h. Replacement of the primary antibody with 0.6% BSA served as a negative control. Sections were washed for 1 min with 20 mM Tris–HCl buffer (2 × 2 min) and stained with 4% w/v aqueous uranyl acetate (VWR International, Lutterworth, UK) for 20 min. The stain was removed with deionized water and sections were air-dried and viewed using a CM12 Transmission Electron Microscope (Phillips Ltd., Cambridge, UK) operating at 80 kV. A total of 24 images were obtained from the tissue sections of three animals within each experimental group. To provide a semi-quantitative measure of TGF-β_1_ levels within the respective healing collagenous matrices, gold-labeled particles were counted within randomly selected fields of view of 30 µm^2^ within each image. The scatter of data was examined to determine mean, median, and quartile ranges. The normality of data was analyzed using the Shapiro–Wilk test. Only data derived from the Young Normal Week 3, Aged Diabetic Week 3, and Young Diabetic Week 9 groups indicated normality. Therefore, data were analyzed using a mixed-parametric one-way ANOVA incorporating the Tukey method (Graphpad Prism statistical software).

### Isolation and extended culture of CB-MSCs

MSCs were isolated by explant culture from the rat compact bone chips (CB-MSCs) obtained from the diaphysis of femur and humerus long bones of 28-day-old male Wistar rats, as previously described (43). Tissue samples were collected in accordance with Basel Declaration guidelines and the UK Animals (Scientific Procedures) Act 1986, following Schedule 1 Code of Practice for the Humane Killing of Animals. Bone marrow was flushed from the long bones and dissected into 1–3 mm^2^ bone chips, which were then digested with a source of collagenase II (sigma-Aldrich) for 2 h at 37°C. Mesenchymal cells from the endosteal and periosteal lining (CB-MSCs) were obtained from explants derived from the culture of the bone chips. CB-MSCs were expanded in complete culture medium (CCM), consisting of αMEM with ribonucleosides and deoxyribonuclosides (ThermoFisher Scientific), 20% heat-inactivated fetal bovine serum (FBS; ThermoFisher Scientific), 1% antibiotics–antimycotics (10,000 units penicillin, 10 mg streptomycin, and 25 µg amphotericin B per ml in original solution; Sigma-Aldrich), and 100 µM L-ascorbic acid 2-phosphate (Sigma-Aldrich), supplemented to achieve normal (5.5 mM) or high (25 mM) glucose concentrations. CB-MSCs were subsequently expanded under normoglycemic or hyperglycemic conditions at 37°C / 5% CO_2_ for approximately 350 days in culture (44). The culture medium was changed every 2–3 days. Population doublings (PDs) were calculated as previously reported, with CB-MSCs expanded to reach PD15 (representing early PDs) or PD150 (late PDs) (44).

### Osteogenic differentiation

CB-MSCs at PD15 and PD150 were seeded at 4,000 cells/cm^2^ and cultured in basal αMEM containing normal (5.5 mM) or high (25 mM) glucose concentrations. At 24 h, the culture media was replaced with respective media supplemented with 100 µM L-ascorbic acid 2-phosphate, 10 nM dexamethasone, and 100 µM β-glycerophosphate (all Sigma-Aldrich). CB-MSCs cultured in the absence of osteogenic factors served as negative controls. At days 2, 7, 14, and 21, the conditioned media and cells were recovered for analysis of gene expression by quantitative PCR (qPCR) or protein levels by western blot analysis (described below). At day 28, cells were fixed with 4% paraformaldehyde solution (Santa Cruz) and mineral-containing nodules visualized by staining with Alizarin red S (Sigma-Aldrich), pH 4.2, for 30 min at room temperature, with images captured by light microscopy (Eclipse TS100 Inverted Microscope; Nikon UK Ltd., Kingston upon Thames, UK).

### M1/M2 macrophage differentiation

A single-cell suspension of bone marrow cells was flushed from the long bones of 4-week-old male Wistar rats using ice-cold PBS by passing it through a 21-gauge needle and then passing it through a 70-µm cell strainer. Cells were centrifuged at 1,800 rpm for 5 min and re-suspended in BD Pharm Lyse™ lysing solution (2 ml; ammonium chloride-based reagent for lysis of red cells; BD Biosciences, Oxford, UK) for 2 min. Viable monocytes were recovered by centrifugation, re-suspended for seeding at 1 × 10^6^ cells/cm^2^ in RPMI 1640 (ThermoFisher Scientific), and supplemented with 10% FBS, 1% antibiotics–antimycotics, and normal (5.5 mM) or high (25 mM) glucose concentrations. RPMI 1640 media was further supplemented with either rhGM-CSF (10 ng/ml) or rhM-CSF (10 ng/ml) and rhIL-4 (10 ng/ml) (all R&D Systems, Abingdon, UK) to promote monocyte differentiation into M1 or M2 macrophages, respectively. Monocytes were maintained at 37°C, 5% CO_2_ for 7 days, with conditioned media collected on days 3 and 5 for analysis of TGF-β_1_ protein levels by ELISA (described below). Cellular morphology was examined by light microscopy at day 7, before harvesting for analysis of M1 and M2 macrophage marker gene expression by qPCR at day 7.

### qPCR

Total RNA was extracted using the RNeasy® Mini Kit and QIAShredder (Qiagen, Manchester, UK). RNA purity and concentration were determined by absorbance at 260 nm/280 nm (NanoVue™; GE Healthcare, Little Chalfont, UK). Complementary DNA (cDNA) was synthesized from 500 ng total RNA, using 5 µl 5× Moloney murine leukaemia virus (M-MLV) buffer, 0.5 µg Random Primers, 0.6 µl RNasin, 1.25 µl deoxynucleotide triphosphates (dNTPs; 10 mM), and 1 µl M-MLV reverse transcriptase, reconstituted in 10 µl nuclease-free water (all Promega, Southampton, UK). cDNA was diluted 1:5 (oseteogenic markers) or 1:10 (macrophage markers) with RNA-free water. qPCR reactions were performed using a 5 µl diluted cDNA sample, 10 µl SYBR Green Precision qRT-PCR Master Mix (Primer Design Ltd., Southampton, UK), 2.5 µl 3 µM primers, and 2.5 µl RNA-free water. Osteogenic and M1/M2 macrophage marker gene primers were purchased from Primer Design Ltd. and Eurofins MWG Operon (Ebersberg, Germany), respectively (Table 1). Reactions were performed using an ABI Prism 7000 Sequence Detection System and ABI Prism 7000 SDS Software V1.0 (ThermoFisher Scientific). All reaction conditions were as follows: 1 cycle of 95°C for 10 min, 40 cycles of 95°C for 15 s, 55°C for 30 s, and 72°C for 30 s. Relative fold changes in marker gene expression (RQ) were calculated using the 2^–ΔΔCt^ method (49), normalized against the β-actin housekeeping gene.

**Table 1:** Primer sequences for quantitative PCR analysis.

Gene	Primer sequence	Length (bp)
Biglycan	F: CCTCCAGCACCTCTATGCTC R: ACTTTGAGGATACGGTTGTC	186
Decorin	F: ACCCGGATTAAAAGGTGGTGA R: TCTCTGCTCAAA TGGTCCAGC	104
TGF-β_1_	F: AAGAAGTCACCCGCGTGCTA R: GGCACTGCTTCCCGAATG	82
Arg1	F:GCAGAGACCCAGAAGAA TGGAAC R:CGGAGTGTTGATGTCAGTGTGAGC	144
IL-6	F:GAGTCACAGAAGGAGTGGCTAA R:ACAGTGAGGAATGTCCACAAAC	146
TNF-α	F:TGTCTGTGCCTCAGCCTCTTC R:TTTGGGAACTTCTCCTCCTTGT	114
iNOS	F:CTTGGAAGAGGAACAACTACTGCT R:GCCAAATACCGCATACCTGAA	139
VEGF	F: ATCATGCGGATCAAACC R: ATTCACATCTGCTATGCT	73
CD163	F: TGTAGTTCATCATCTTCGGTCC R: CACCTACCAAGCGGAGTTGAC	97
Osterix	F: GCTTTTCTGTGGCAAGAGGTTC R: CTGATGTTTGCTCAAGTGGTCG	136
Osteopontin	F: TCCAAGGAGTATAAGCAGAGGGCCA R: CTCTTAGGGTCTAGGACTAGCTTGT	200
Osteocalcin	F: ACAGACAAGTCCCACACAGCAACT R: CCTGCTTGGACATGAAGGCTTTGT	161
β-actin	F: TGAAGATCAAGATCATTGCTCCTCC R: CTAGAAGCATTTGCGGTGGACGATG	155

### Western blot analysis

Conditioned media was collected at days 2, 7, 14, and 21, centrifuged at 8,000 rpm for 5 min, and stored at −80°C, until required. At these same time points, adherent cells were washed in ice-cold PBS (×2) and cellular-associated protein extracted by treatment with 0.1% Triton X-100, 0.05 M sodium acetate buffer, pH 6.8, containing cOmplete™ Protease Inhibitor Cocktail (Roche, Burgess Hill, UK) at 37°C/5% CO_2_ for 15 min. The residual ECM was washed in ice-cold PBS (×2) before treatment with 2% Triton X-100, 4 M guanidinium hydrochloride, 0.05 M sodium acetate buffer, pH 6.8, containing cOmplete™ Protease Inhibitor Cocktail at 37°C/5% CO_2_ for 15 min. Conditioned media, cell lysates, and ECM extracts were exhaustively dialyzed against double-distilled water, containing protease inhibitors, before lyophilization. Extracts were re-suspended in PBS and protein concentrations quantified (Pierce® BCA Protein Assay Kit; ThermoFisher Scientific).

Protein samples (20 µg) were separated under reducing conditions by sodium dodecyl sulfate–polyacrylamide gel electrophoresis (SDS–PAGE) on pre-formed 4%–15% Mini-PROTEAN**®** Precast Gels (Mini-Protean® Tetra Cell System; BioRad, Hemel Hempstead, UK) and electroblotted onto polyvinylidene difluoride membranes (Hybond™-P; ThermoFisher Scientific) using a Semi-Dry Trans-Blot System (BioRad), as per the manufacturer's instructions. Membranes were blocked with 5% semi-skimmed milk/1% Tween 20 in Tris-buffered saline (TBS) at 4°C overnight. Membranes were immuno-probed with primary antibodies specific to TGF-β_1_ (polyclonal anti-goat, 1:100; Santa Cruz), total Smad 2/3 (mouse monoclonal anti-rabbit, 1:1,000; New England Biolabs, Hitchin, UK), and using mouse monoclonal IgG against the core protein of decorin (70.6) and biglycan (PR8A4) (both used at 1:50; gifts from Professor Bruce Caterson, School of Biosciences, Cardiff University, UK), diluted in 5% semi-skimmed milk/1% Tween 20 at room temperature for 1 h. Membranes were washed (×3) in 1% TBS-Tween and incubated with either HRP-conjugated, rabbit anti-goat IgG (to detect TGF-β_1_, 1:3,000; Santa Cruz), HRP-conjugated, rabbit anti-mouse IgG (to detect decorin or biglycan, total Smad 2/3, 1:50,000; Abcam), or HRP-conjugated, polyclonal swine anti-rabbit IgG (to detect β-actin Loading Control, 1:3,000; Dako UK Ltd., Cambridge, UK). Secondary antibodies were diluted in 5% semi-skimmed milk/1% Tween 20 for 1 h at room temperature. Membranes were washed (×3) in 1% TBS-Tween and TBS and incubated in ECL™ Prime Detection Reagent (VWR International) and autoradiographic films (Hyperfilm™-ECL; ThermoFisher Scientific) developed as per the manufacturer's instructions.

### Enzyme-linked immunosorbent assay (ELISA)

Conditioned media was collected during monocyte differentiation into the M1 or M2 macrophages under normoglycemic and hyperglycemic conditions at days 3 and 5, as described above. TGF-β_1_ concentrations in the conditioned media were quantified using TGF-β_1_ Platinum ELISA Kits (eBioscience, Hatfield, UK), as per the manufacturer's instructions. Absorbance values were measured using a FLUOstar® Optima Microplate Reader (BMG Labtech, Aylesbury, UK) at 450 nm, with data expressed as pg/ml.

### Statistical analysis

Data are expressed as mean ± standard deviation (SD). The normality of data was confirmed using the Shapiro–Wilk test. The statistical significance of the differences between the experimental groups was evaluated using a parametric one-way ANOVA, followed by multiple comparisons using Tukey's test with Graphpad Prism statistical software. All experiments were performed in triplicate and repeated on three separate occasions.

## Results

### *In vivo* bone repair and TGF-β_1_ sequestration during implant osseointegration

**Figure 1:**
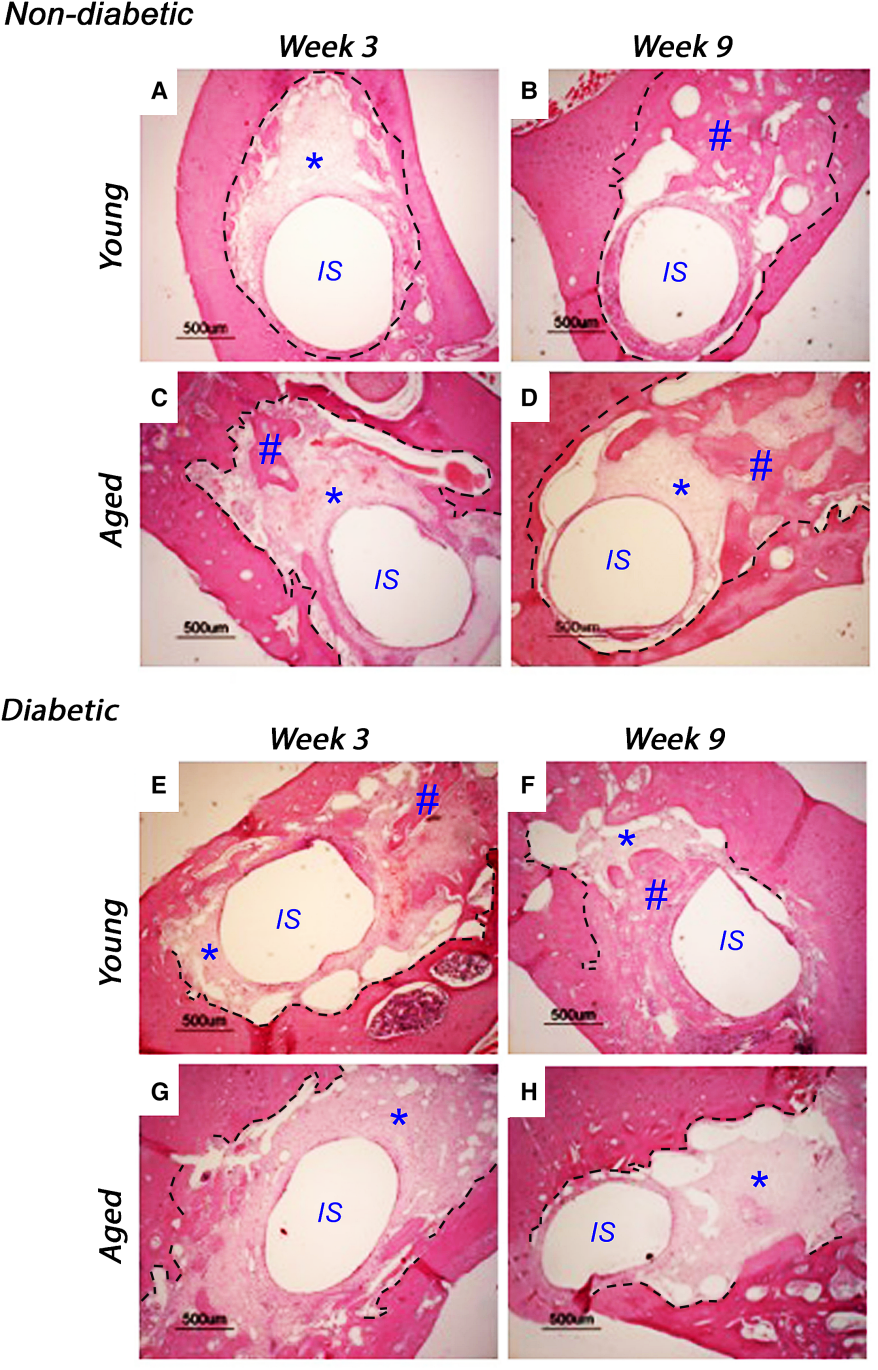
Representative histological images of tissue sections stained with hematoxylin and eosin, monitoring bone healing around implants placed into the mandibles of 10-week-old (young) and 18-week-old (aged) diabetic male Goto-Kakizaki (GK) rats and age-matched, non-diabetic male Wistar rat controls, at 3 weeks (**A**, **C**, non-diabetic; **E**, **G**, diabetic) and 9 weeks (**B**, **D**, non-diabetic; **F**, **H**, diabetic) after implant insertion. Tissue sections transverse to the implant were collected from three animals per experimental group. Scale bar = 500 µm. The central region, labeled IS, represents the original site of implant removal. The broken lines define identifiable regions for old and new bone as identified by boundaries for granulation tissue and evident reversal lines. * indicates sites of granulation tissue; # indicates sites of new bone formation.

Implants were inserted into freshly excised tooth sockets of 10-week-old (young) and 18-week-old (aged) diabetic male Goto-Kakizaki (GK) rats and age-matched, non-diabetic male Wistar rats; bone deposition was monitored using histological analysis (Figure 1). At 3 weeks after insertion, the non-diabetic (control) tissues of the young (Figure 1A) and aged (Figure 1C) animals exhibited cell-rich, granulation tissue formation around the implant insertion sites, with additional evidence of distance osteogenesis, identifiable as the formation of new bone-like tissue extending from the original bone tissue of the tooth sockets. At 9 weeks after insertion, new bone formation was noted to have progressed in both the non-diabetic, young (Figure 1B) and aged (Figure 1D) tissues, although the deposition of fully mineralized bone was observed to be more advanced in the younger tissues, with greater contact osteogenesis evident close to the implant surface, compared to the aged animals. The new bone was predominantly cancellous bone, which precluded accurate measurement of new bone formation by image analysis.

When comparing the young diabetic animals (Figures 1E,F) with their age-matched non-diabetic equivalents (Figures 1A,B), the rate of bone deposition was slightly reduced by the hyperglycemic wound-healing environment, with greater levels of mineralized bone via distance osteogenesis evident within the young non-diabetic group. However, and in stark contrast, significant areas of soft granulation tissue were still evident at 3 and 9 weeks after implant insertion with very little contact osteogenesis apparent within the aged diabetic rats (Figures 1G,H), indicating that bone healing was significantly impaired compared to both the aged normal and the young diabetic animals.

**Figure 2:**
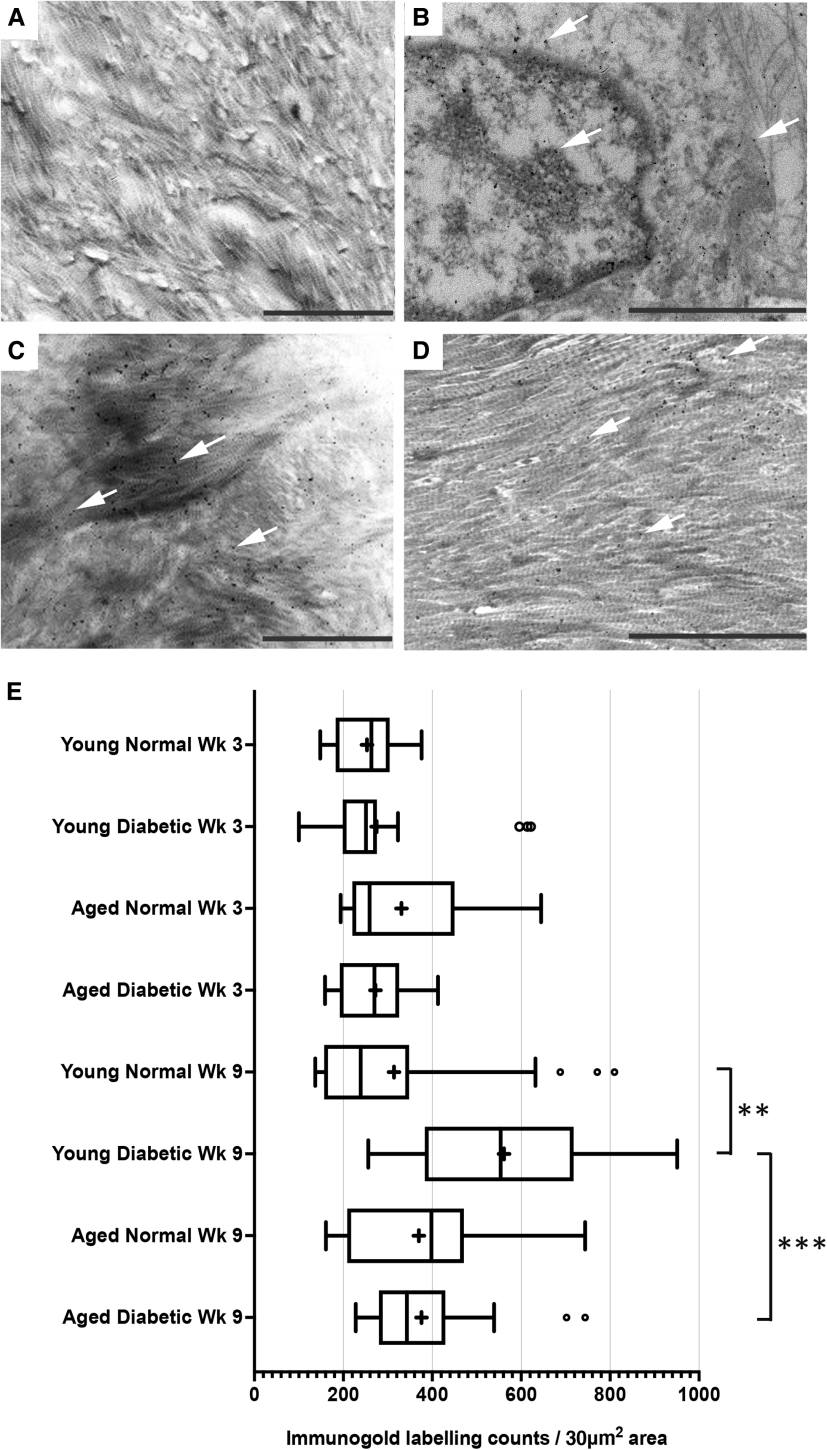
Immunogold labeling and semi-quantification of TGF-β_1_ within tissue sections obtained from healing bone matrices approximating near implant placement sites. Shown are representative images to indicate examples for labeling (white arrows) found in all groups. (**A**) Control section indicating no gold labeling within areas rich in forming collagen fibers (primary antibody omitted). (**B**) Immuno-labeling for TGFβ_1_ associated with cells and the pericellular regions near the implant site. (**C**,**D**) Immunolocalization of TGF-β_1_-associated newly forming collagen fibers. Scale bar = 2 µm. (**E**) Semi-quantification of TGF-β_1_ immuno-labeling in diabetic and non-diabetic experimental groups. Data are presented as a box and whisker plot (*n* = 24), presenting upper and lower extremes of the data with outliers omitted as determined by the Grubbs test. The boxes outline the upper and lower quartiles, mean (marked by +) and median (marker by I within the box). ***p < 0.001, **p < 0.01.

After visualization by TEM, representative images of immunogold labeling for TGF-β_1_ obtained from reparative tissues close to the implant sites are shown in Figure 2. Antibody-free controls exhibited no gold labeling (Figure 2A). Initial observations showed that immunogold labeling, representing TGF-β_1_ sequestration, was predominately associated with the pericellular regions (arrows, Figure 2B) and collagen fibers throughout the healing sites (arrows, Figures 2C,D). However, labeling did not conform to any regular patterning that matched the banding of the collagen fibers. Similar staining patterns were obtained for all experimental groups.

A semi-quantitative analysis of TGF-β_1_ levels within the respective healing collagenous matrices was calculated and presented as box and whisker plots (Figure 2E). The data obtained demonstrated moderately widespread particle counts overall, suggesting an uneven distribution of labeling throughout the healing matrices. As the data point distributions did not exhibit normality for all groups, a Kruskal–Wallis non-parametric ANOVA analysis with Dunn's test for multiple comparisons was performed, which identified no significant differences between diabetic and normal, or aged and young tissues at 3 weeks after implant insertion (all *p* *>* 0.05) (Figure 2E). However, significant increases in immuno-labeling levels for TGF-β_1_ sequestered within the healing matrices of young diabetic tissues at 9 weeks after implant insertion were observed, compared to the TGF-β_1_ immuno-labeling counts in aged and non-diabetic tissues (all *p <* 0.01) (Figure 2E).

### High glucose has negligible effect on cell expansion but a negative impact on the osteogenic differentiation of CB-MSCs *in vitro*

The present study used CB-MSCs, where long-term expansion in normoglycemic conditions has previously been characterized to indicate that isolated cell populations, expanded to PD15 in culture in a normoglycemic basal media, contain a high population of low colony-forming, pre-osteoblast cells that contribute to the bone lining cell population (44). However, following expansion beyond PD50, the heterogeneous cell population becomes dominated by high colony-forming, multipotent cells (44). Within the present study, long-term cultures in normoglycemic (5.5 mM) and hyperglycemic (25 mM) conditions were demonstrated to have a limited impact on CB-MSC expansion capabilities over 350 days in culture. CB-MSCs expanded in normoglycemic conditions took 156 days to reach PD150, while in hyperglycemic conditions, CB-MSCs took a further 12 days (178 days). Analyses were performed on CB-MSCs at PD15 and PD150 to allow some comparison with *in vivo* findings, as CB-MSCs in the GK rats were exposed to a hyperglycemic environment for 10–27 weeks (189 days).

**Figure 3:**
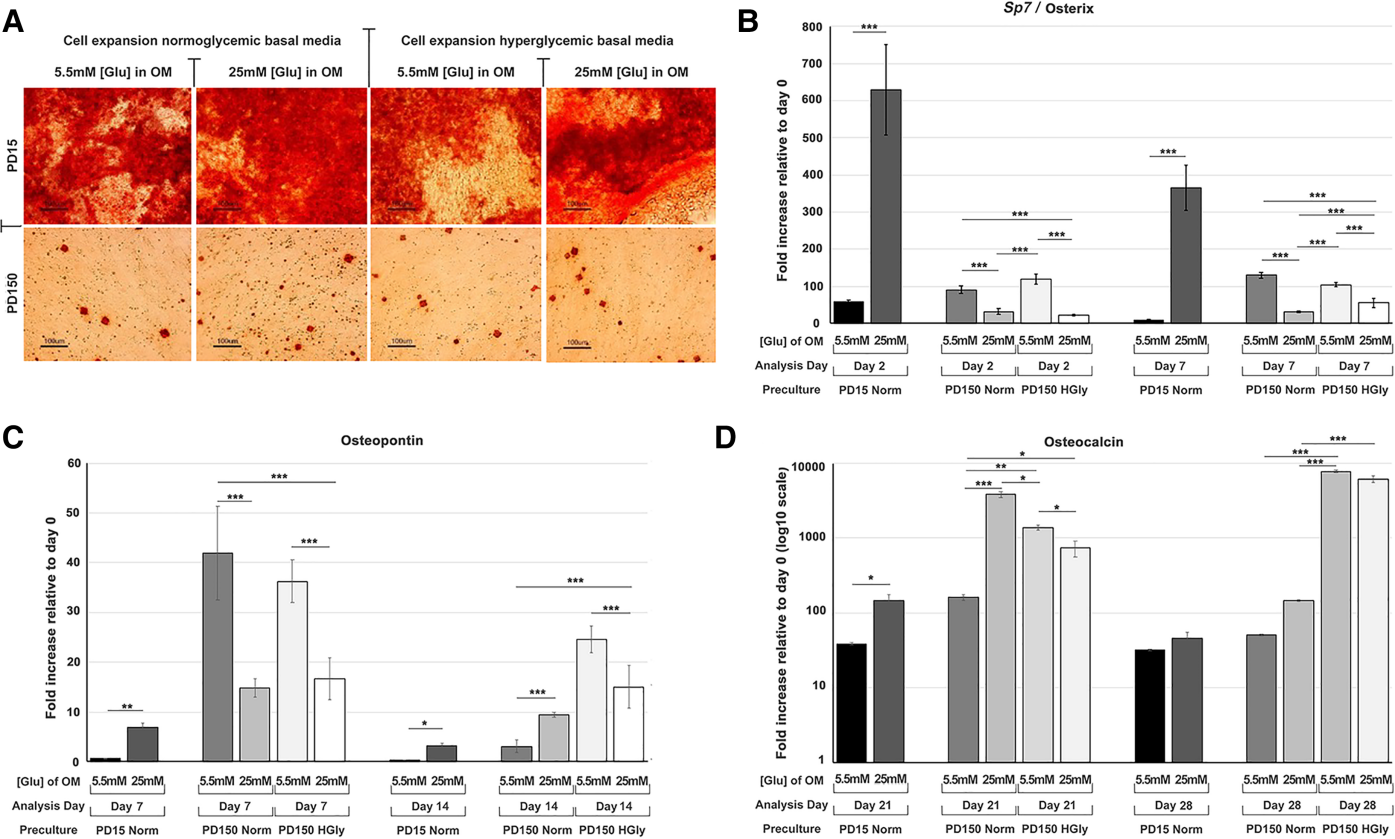
Osteogenic differentiation of CB-MSCs expanded to PD15 under normoglycemic conditions (5.5 mM glucose osteogenic medium, PD15 Norm), PD15 under hyperglycemic conditions (25 mM glucose osteogenic medium, PD15 HyGly), PD150 under normoglycemic conditions (5.5 mM glucose osteogenic medium, PD150 Norm), and PD150 under hyperglycemic conditions (25 mM glucose osteogenic medium, PD150 HyGly). (**A**) Representative images of Alizarin red staining demonstrating mineralized matrix synthesis by CB-MSCs expanded to PD15 or PD150 under normoglycemic and hyperglycemic conditions, following which osteogenesis was induced for 28 days in media containing normal (5.5 mM) or hyperglycemic (25 mM) glucose conditions. (**B–D**) Measured effects of high glucose exposure on the expression of early osteogenic marker, *Sp7*/osterix, mid-stage osteogenic marker, osteopontin, and late osteogenic marker, osteocalcin. Values were normalized to the expression of β-actin and presented as mean ± SD fold change, relative to day 0 (note that osteocalcin is presented on a log_10_ scale, due to the large fold differences observed). *n* = 3, *p *<* 0.05, **p *<* 0.01, ***p *<* 0.001. *p*-values were calculated using one-way ANOVA and Tukey's multiple comparison post-test. PD, population doubling.

Within the present study, we provided further data relating to the effects of high glucose (25 mM) supplementation on osteogenic differentiation and associated marker gene expression by CB-MSCs (Figure 3). When considering the effects of short-term exposure to high glucose concentrations on CB-MSCs originally cultured to PD15 under normoglycemic conditions, subsequent culture in osteogenic media supplemented with 25 mM glucose resulted in significant increases in the expression of *Sp7*/osterix (Figure 3A), along with lesser, yet significant increases in gene expressions of osteopontin (Figure 3B) and osteocalcin (Figure 3C), which correlated with a moderate increase in Alizarin red staining (Figure 3D). For CB-MSCs cultured to PD150 under normoglycemic conditions, subsequent culture in osteogenic media supplemented with 25 mM glucose resulted in significant decreases in *Sp7*/osterix (Figure 3A) and osteopontin (Figure 3B) but an increase in osteocalcin (Figure 3C). There appeared to be little further effect on mineral nodule formation (Figure 3D), which was already limited due to long-term culture expansion, leading to a predominance of transit-amplifying cells that would be expected to take longer to differentiate *in vitro*. Similar results were observed for CB-MSC cultures expanded in hyperglycemic conditions, where continued culture of these cells in hyperglycemic osteogenic induction media caused a decrease in *Sp7*/osterix (Figure 3A) and osteopontin (Figure 3B) and a slight delay in osteocalcin gene expression (Figure 3C) when compared to CB-MSCs culture expanded in hyperglycemic but then cultured in 5.5 mM normoglycemic conditions during osteogenic induction. Likewise, the inclusion of high glucose during osteogenic induction of PD150 expanded in hyperglycemic conditions elicited little further effect on nodule formation, which was low due to culture expansion.

### Hyperglycemic conditions induced perturbations in TGF-β_1_ expression and secretion *in vitro*

**Figure 4:**
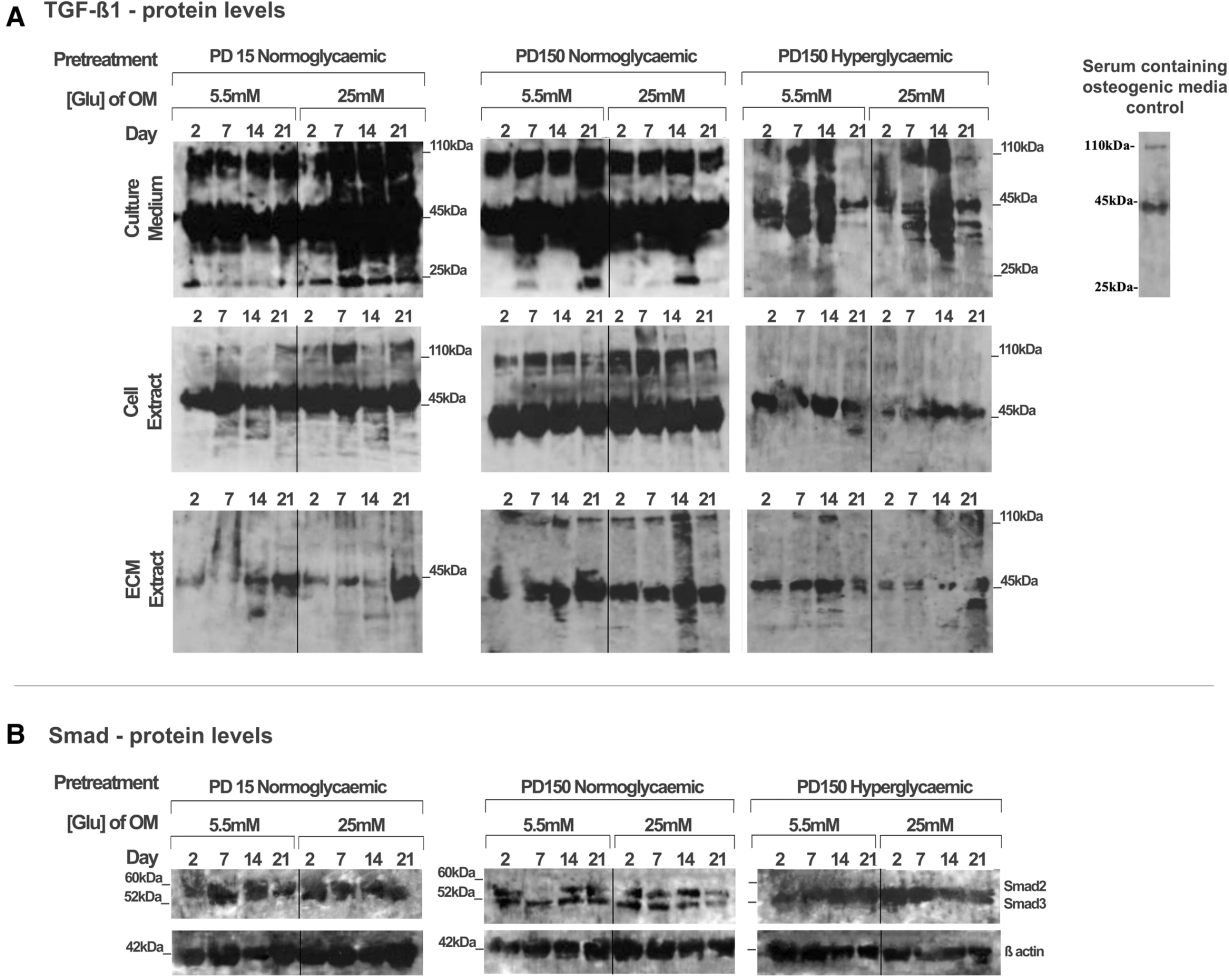
TGF-β_1_ synthesis, secretion, and distribution during the osteogenic differentiation of CB-MSCs expanded to PD15 under normoglycemic conditions (5.5 mM glucose osteogenic medium), PD150 under normoglycemic conditions (5.5 mM glucose osteogenic medium), and PD150 under hyperglycemic conditions (25 mM glucose osteogenic medium). (**A**) Representative western blot images of TGF-β_1_-associated protein localization in CB-MSC culture media, cell lysates, and ECM extracts over 21 days in osteoinductive culture. (**B**) Representative western blot images of total Smad2/3 levels associated with TGF-β_1_ signaling, in cell lysates of CB-MSCs at PD15 or PD150 under normoglycemic or hyperglycemic conditions. For all western blots presented, protein was loaded at 20 µg to allow the visualization of all TGF-β_1_ isoforms: ∼55 kDa pre-pro-TGF-β_1_ monomers, the ∼45 kDa precursor protein, TGF-β_1_ precursor attached to LAP, ∼110 kDa TGF-β_1_-LAP homodimers, and 25 kDa active TGF-β_1_ dimer. ECM, extracellular matrix; OM, osteogenic medium; PD, population doubling.

Using the CB-MSC populations that had initially been expanded to PD15 or PD150 in either normoglycemic or hyperglycemic basal media, the subsequent effects of high glucose concentrations on TGF-β_1_ during osteogenic induction were analyzed, examining levels localized intracellularly, sequestered within the ECM, or released into the culture medium (Figure 4). When considering the bioavailability and the signaling activity of TGF-β_1_, it is important not only to assess the levels of growth factor but also to identify the different isoforms. Western blot analysis facilitated the detection of TGF-β_1_ in several processed isoforms: the ∼55 kDa pre-pro-TGF-β_1_ monomers; the ∼45 kDa precursor protein; and, when attached to its latency associated protein (LAP), the ∼110 kDa TGF-β_1_-LAP homodimers; and the 25 kDa active TGF-β_1_ dimer (19). For most blots, the inactive ∼55 kDa pre-pro-TGF-β_1_ monomers and the ∼45 kDa precursor protein predominated. In order to identify the 25 kDa active dimer and the TGF-β_1_ form attached to LAP, it was necessary to load gels with a high level of protein (20 µg). This did have the consequence of producing blots where protein bands with high intensity could not be measured by densitometry due to their supersaturated pixel density, which would produce false results. Further, due to the high number of samples analyzed, the detection of TGF-β_1_ was necessarily performed on a separate gel and, although protein-loading levels were equal for each gel, this hampered any direct quantification when comparing groups. However, a comparison of the respective blots still presents clear observations for monitoring changes in the levels of the various iosforms. The ∼55 kDa pre-pro-TGF-β_1_ monomers and the ∼45 kDa precursor protein were detected in the cell lysate, incorporated into the ECM, and released into the culture media by CB-MSCs during the osteoinduction of CB-MSCs expanded to PD15 in normoglycemic basal media (Figure 4A). The addition of 25 mM glucose to the osteoinduction media had little effect on the levels of the TGF-β_1_ monomer/precursor protein, but a substantial increase in the level of the 110 kDa TGF-β_1_-LAP homodimers was observed in the protein isolated from the cell extract and that released into the culture media (Figure 4A).

Long-term expansion of CB-MSCs to PD150 in normoglycemic conditions indicated that the TGF-β_1_-processed isoforms, synthesized following osteogenic differentiation under normoglycemic conditions, were generally comparable to CB-MSCs at PD15, with the immuno-detection of the TGF-β_1_ monomer (∼45–55 kDa) and TGF-β_1_-LAP homodimers at ∼110 kDa within the cell lysates, the associated ECMs, and the culture media. When these normoglycemic PD150 CB-MSCs were driven down an osteogenic lineage under high glucose conditions, few changes in the TGF-β_1_ isoforms forms were noted (Figure 4A). However, long-term CB-MSC expansion under hyperglycemic conditions to PD150 led to significant reductions in detectable TGF-β_1_ overall, with the notable loss of detectable TGF-β_1_ at ∼110 kDa in the cell and ECM extracts and overall loss of the active TGF-β_1_ bands at ∼25 kDa (Figure 4A). Such responses were further exacerbated when these CB-MSCs were cultured in hyperglycemic (25 mM glucose) osteoinductive medium, with further reductions in cell-, ECM-, and culture medium-associated TGF-β_1_ immuno-reactivity observed (Figure 4A).

To corroborate our observed results, the gene expression for TGF-β_1_ was also examined using qPCR (see Supplementary Material). The results, however, suggested overall increases in TGF-β_1_ gene expression after the long-term expansion of CB-MSCs in normoglycemic basal culture media to PD150, and smaller increases in TGF-β_1_ were observed for CB-MSCs expanded to PD150 in hyperglycemic media. Thus, although increases in TGF-β_1_ gene expression were observed, the subsequent translation and synthesis of TGF-β_1_ proteins did not appear to be evident. In considering our research aim for investigating the bioavailability and bioactivity of TGF-β_1_, the focus was therefore placed on the results obtained for the analysis of protein levels within the extracellular matrix and culture media.

To assess whether the signaling potential of TGF-β_1_ was disrupted in CB-MSCs subjected to such contrasting levels of hyperglycemic exposure, we also evaluated the impact on total Smad2 and Smad3 expression levels in considering their roles as key regulators of TGF-β_1_ signaling (50, 51). Both Smad proteins were detectable in the cell lysates obtained from CB-MSCs (Figure 4B), but long-term CB-MSC expansion, whether in normo- or hyperglycemic conditions, was observed to have minimal effects on total Smad2/3 levels.

### Short- and long-term exposure of CB-MSCs to high glucose affects the synthesis and secretion of decorin and biglycan *in vitro*

**Figure 5:**
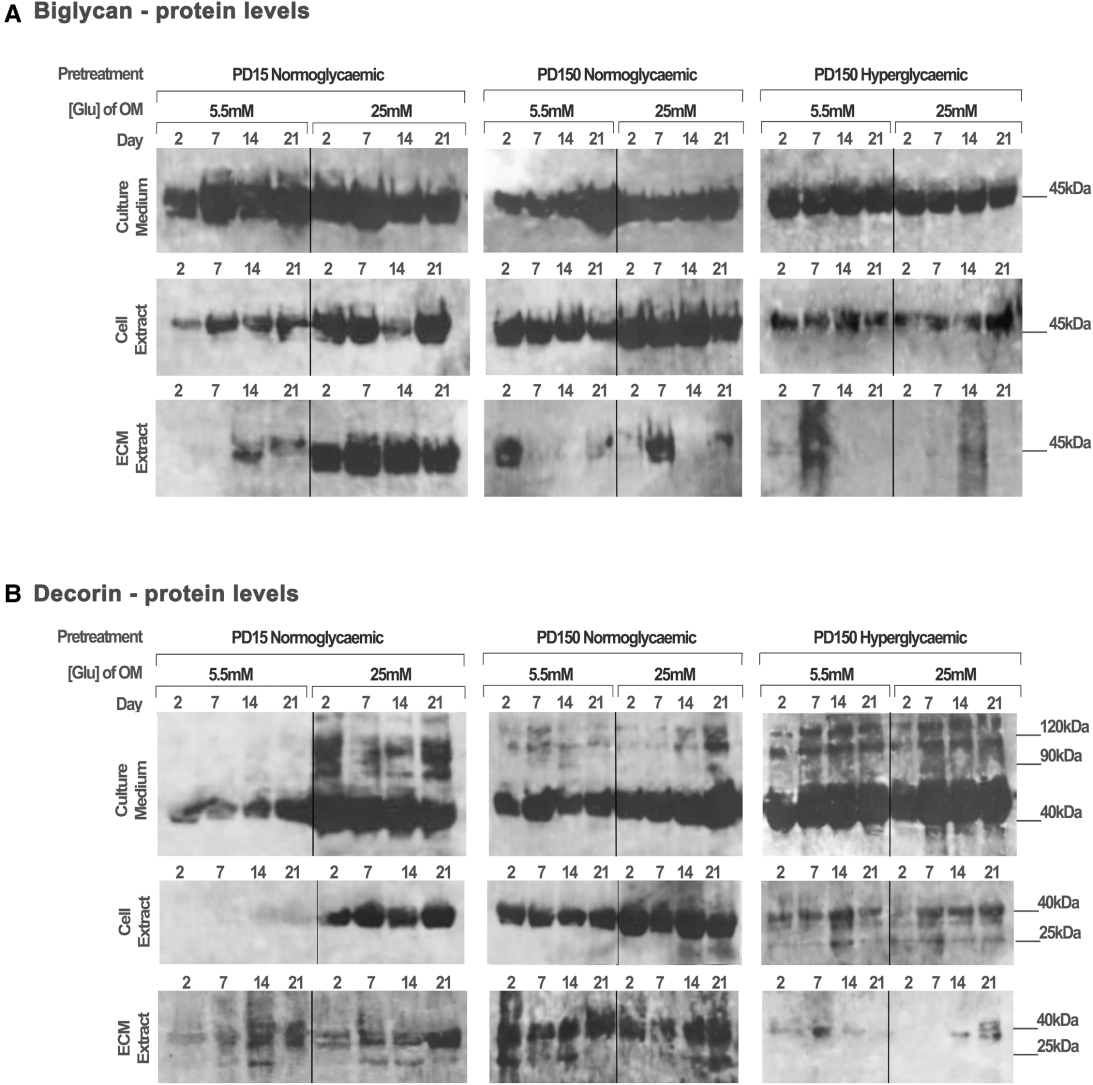
Biglycan and decorin synthesis, secretion, and distribution during the osteogenic differentiation of CB-MSCs expanded to PD15 under normoglycemic conditions (5.5 mM glucose osteogenic medium), PD150 under normoglycemic conditions (5.5 mM glucose osteogenic medium), and PD150 under hyperglycemic conditions (25 mM glucose osteogenic medium). (**A**) Representative western blot images of biglycan protein localization in CB-MSC culture media, cell lysates, and ECM extracts over 21 days in culture. (**B**) Representative western blot images of decorin protein localization in CB-MSC culture media, cell lysates, and ECM extracts over 21 days in culture. For all western blots presented, protein was loaded at 20 µg to allow a visual comparison with western blots detecting TGF-β_1_ and the identification of degradation products of decorin and biglycan noted at 25 kDa. ECM, extracellular matrix; OM, osteogenic medium; PD, population doubling.

Protein extracts from the CB-MSC populations analyzed above were also assessed to identify the effects of high glucose (25 mM) on the protein levels of decorin and biglycan, as potential binding partners for TGF-β_1_ (Figure 5). Western blot gels were loaded with the same amount of protein (20 µg) to allow for comparison with the TGF-β_1_ levels reported above and to identify processed products that would influence their ligand binding properties. As discussed above, this prevented a quantitative analysis but did provide observational trends in the data. Immuno-detection for biglycan produced strongly staining bands at ∼45 kDa, which correspond to its core protein, while immuno-detection for decorin synthesized and secreted by the CB-MSC populations revealed strongly staining bands at ∼45 kDa and ∼120 kDa, corresponding to the molecular weights of the decorin core protein and decorin conjugated to its glycosaminoglycan chain, respectively (52).

When analyzing CB-MSCs at PD15, supplementation of the osteogenic induction media with 25 mM glucose led to a moderate increase in biglycan immuno-detection within the cell lysate and the ECM extract (Figure 5A). High levels of biglycan were released into the culture media by PD15 CB-MSCs; however, there were no discernible differences in the levels observed when comparing cells cultured in normo- or hyperglycemic osteogenic induction media (Figure 5A). For these same cells, the presence of high glucose in the osteogenic induction media resulted in considerable increases in decorin extracted from the cell lysate and the culture media, but few differences were identifiable in the levels incorporated into the ECM extract (Figure 5B).

The long-term expansion of CB-MSCs to PD150 in either normoglycemic or hyperglycemic culture conditions resulted in subtle decreases in the levels of biglycan core protein in the cell culture media (Figure 5A). However, the long-term expansion of CB-MSCs in normoglycemic conditions indicated a moderately increased level of biglycan detected in the cell lysate of CB-MSCs after culture expansion in normoglycemic conditions, but reduced levels of biglycan in cell lysates were observed after culture expansion in hyperglycemic culture media (when compared to PD150 in normoglycemic conditions). Low levels of biglycan were incorporated into the ECM matrix whether expanded in normo- or hyperglycemic conditions.

The immuno-detection of decorin after long-term culture expansion presented different results (Figure 5B). Culture expansion of CB-MSCs to PD150 in normoglycemic conditions was associated with moderately increased levels of decorin isolated from the cell lysate, ECM, and culture media. Supplementation of the osteoinductive media with 25 mM glucose increased the levels of decorin detected in the culture media and cell lysate further. When CB-MSCs were cultures expanded to PD150 in hyperglycemic conditions, similar increases in decorin were noted in the culture media and cell lysate, but little decorin was incorporated into the ECM. The inclusion of high glucose in the osteoinductive media had little further discernable influence on decorin levels contained within any of the protein extracts from the cell, ECM, or released into the culture media (Figure 5B).

These cells were also analyzed using qPCR to examine the effects of high glucose on the gene expression of decorin and biglycan by the cells after culture expansion and the presence of high or normal glucose levels (Supplementary Material). These results partially corroborated with the raised protein levels for decorin and biglycan reported above.

### Short-term hyperglycemic conditions hinder the formation of the M2 macrophage phenotype

**Figure 6:**
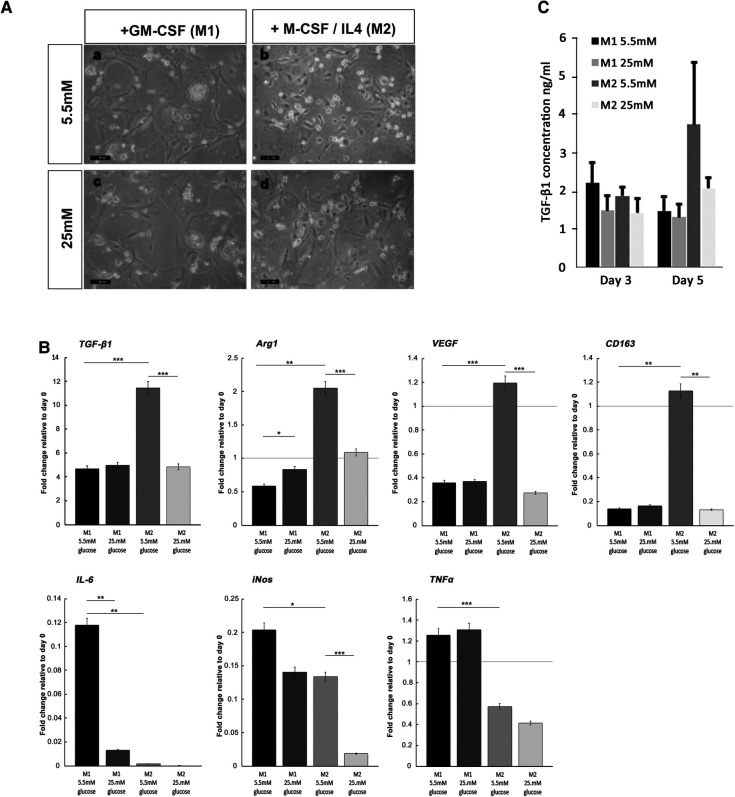
Monocyte differentiation into M1 and M2 macrophages under normoglycemic conditions and hyperglycemic conditions. (**A**) Representative images of M1 and M2 macrophage morphology, produced from monocytes stimulated with either GM-SCF (to produce the M1 phenotype) or M-CSF/IL-4 (to yield the M2 phenotype) in either normal (5.5 mM glucose) or high (25 mM glucose) medium. (**B**) ELISA quantification of TGF-β_1_ levels produced by differentiating M1 and M2 macrophages, at days 3 and 5 in either normal (5.5 mM glucose) or high (25 mM glucose) medium. (**C**) Quantitative PCR analysis for the expression of marker genes associated with the M1 macrophage (IL-6, iNOS, and TNF-α) and M2 macrophage (TGF-β_1_, Arg1, VEGF, and CD163) phenotypes, after 7 days in either normal (5.5 mM glucose) or high (25 mM glucose) medium. Values were normalized to β-actin expression and presented as mean ± SD fold change. For all analyses, *n* = 3, *p *<* 0.05, **p *<* 0.01, ***p *<* 0.001. *p-*values were calculated using one-way ANOVA and Tukey's multiple comparison post-test.

For both macrophage phenotype induction conditions in basal (5.5 mM) glucose concentrations, full macrophage differentiation was achieved after 7 days, as judged by the development of a heterogeneous population containing round or oval cell morphologies (Figure 6A). The effect of high glucose conditions on the formation of the M1 or M2 phenotype was subsequently examined by the quantification of established M1 and M2 macrophage marker genes using qPCR analysis (44, 45). In accordance with these prior published data, M1 macrophage formation in GM-CSF-stimulated cultures exhibited significant increases in the gene expression of pro-inflammatory cytokines, TNF-α (*p* *<* 0.001) and IL-6 (*p* *<* 0.01), in addition to inducible nitric oxide synthase (iNOS; *p* *<* 0.05) expression, compared to isolated monocytes at day 0 (Figure 6C). However, under hyperglycemic conditions, significant decreases in IL-6 (*p* *<* 0.01) were observed, although TNF-α and iNOS expressions were unaffected (*p* *>* 0.05). In contrast, after M-CSF/IL-4 induction, M2 macrophages displayed an increased expression of classical markers TGF-β_1_ (*p* *<* 0.001), arginase 1 (Arg1; *p* *<* 0.01), vascular endothelial growth factor (VEGF; *p* *<* 0.001), and CD163 (*p* *<* 0.01) compared to isolated monocytes at day 0 (Figure 6C). When M2 induction was performed in high (25 mM) glucose conditions, the expression of each of these markers was significantly downregulated (*p* *<* 0.001 for TGF-β_1_, Arg1, and VEGF; *p* *<* 0.01 for CD163), suggesting the significant attenuation of M2 macrophage formation by hyperglycemic environments.

The quantification of TGF-β_1_ released into the culture media by differentiating M1 and M2 macrophages by ELISA demonstrated that secreted TGF-β_1_ levels rose significantly for developing M2 macrophages by day 5 in culture (Figure 6B). The presence of high glucose (25 mM) in the M2 inductive media resulted in a reduction in TGF-β_1_ secretion to basal levels that were consistent with secretions from M1 developing macrophages. It is noted that these findings on the secreted levels of TGF-β_1_ were corroborated by the significant decreases in TGF-β_1_ gene expression (Figure 6C).

## Discussion

Within this study, we present new information that considers the influence of hyperglycemic environments on the bioavailability and activities of TGF-β_1_ within normal and diabetic healing bone. Using a non-obese GK rat *in vivo* model of T2DM (11–14), the histological assessment of tissue healing around inserted implants confirmed delayed bone repair in diabetic, aged, and diabetic aged animals. However, immuno-labeling for TGF-β_1_ was only seen to be significantly increased in healing collagenous matrices of young diabetic animals, compared to young and aged normal Wistar and aged GK rats. In considering the source of TGF-β_1_ within the healing matrix, *in vitro* studies performed on CB-MSC populations demonstrated that short-term exposure to high glucose levels increased the synthesis and secretion of TGF-β_1_ and the regulatory SLRP, decorin, for cells at low population doublings (PD15), but changes were less discernible at high population doublings (PD150), suggesting that the cell response is determined by their differential status. Conversely, after the long-term exposure of CB-MSCs to high glucose concentrations (over approximately 350 days), TGF-β_1_, decorin, and biglycan levels were significantly diminished, although these CB-MSCs retained proliferative capabilities and did not undergo cellular senescence (43, 44).

The *in vivo* studies presented herein provide an extension to those we have previously reported, where semi-quantitative data following immuno-labeling for TGF-β_1_ indicated raised levels of the growth factor at the later stages of bone healing, which has the potential to delay the full osteoblast differentiation and hence the deposition of a mineralized bone matrix (12). Within the present study, we used immunogold labeling, which was able to better quantify the TGF-β_1_, due to the stochiometric interaction of the primary antibody with the secondary gold-labeled antibody. The results confirmed our previous findings identifying raised levels in young diabetic healing osseous tissue (12), but also identified that this was not the case for aged non-diabetic and diabetic animals. Considering the role of TGF-β_1_ in mesenchymal condensation and early commitment of MSCs toward osteogenic differentiation, our observed results could be attributed to the reduced proliferative and hence regenerative capacity of MSCs noted in aged tissues (17). Through immunogold labeling, we were also able to note that TGF-β_1_ was distributed diffusely throughout the healing tissue and did not appear to be bound to static structures, such as collagen fibers, suggesting that the growth factor was potentially more freely available to interact with cell surface receptors when present in active forms free of its ligand binding partners of decorin and biglycan (29, 30).

We have previously published data characterizing the CB-MSCs used in this study, which were isolated from the endosteal and periosteal regions of compact bone (43, 44). These studies assessed colony-forming efficiency, cell cycle proteins, and efficacy for forming a mineralized matrix and indicated that CB-MSCs at PD15 contained a low proliferative pre-osteoblast population capable of responding quickly to repair processes. As *in vitro* cell expansion continued to PD150, the highly proliferative, transit-amplifying cells became the dominant cell population in the culture, which were able to maintain their immature stem cell properties and did not acquire a senescent phenotype (44). Within the correct signaling milieu of bone-healing sites, these immature MSCs are directed to initially proliferate and then differentiate into osteoblasts, before offering the capacity to synthesize a mineralized matrix (2, 3). To confirm previous findings, we identified that mineralized matrix deposition occurs earlier for CB-MSCs at PD15 compared to cells at PD150 (43, 44). We also confirmed that increasing glucose concentrations in osteogenic medium or expanding cells to PD150 in hyperglycemic basal media exerted inhibitory effects on the ability of the cells to deposit a mineralized bone matrix. However, we additionally identified that for CB-MSCs at PD15, osterix expression in high glucose osteogenic induction medium was increased, suggesting an ability for osteoblasts to achieve terminal differentiation (53). However, osteopontin expression was also increased, and recognizing its role in inhibiting mineral crystal growth (1) may contribute to a reduced deposition of a mineralized matrix. In contrast, for CB-MSCs at PD150 expanded in the presence or absence of hyperglycemic conditions, increased osteopontin expression was coupled with reduced osterix expression, which would indicate that high glucose hindered the commitment of transit-amplifying cells to the osteoblast lineage. For both cell populations containing high glucose in either the basal or osteogenic media, disturbances in the timing and levels of osteocalcin expression were also observed, suggesting disorder in the deposition of an ECM facilitating mineral deposition (54).

In performing its role, TGF-β_1_ acts via Smad-dependent pathways to influence the synthesis of essential osteogenic transcriptional and matrix proteins, including osterix, type 1 collagen, and osteocalcin (3, 19). Thus, it may be hypothesized that TGF-β_1_ may, in part, be responsible for the hyperglycemia-induced perturbations in matrix production apparent in CB-MSCs, as described above. However, it is noteworthy that the analysis of intracellular levels of Smad2/3 was minimally affected, suggesting that TGF-β_1_ signaling pathways remained potentially viable, although it is acknowledged that signaling is also regulated by inhibitory Smads and can also proceed via non-canonical routes (22).

Central to this study was the assessment of the effects of short- and long-term exposure to high glucose on the synthesis and secretion of TGF-β_1_, and its matrix ligands of decorin and biglycan, by CB-MSCs at PD150, containing predominantly transit-amplifying cells, compared to PD15, containing a population of MSCs committed to the osteoblast lineage. Previous studies have proposed that the ability of TGF-β_1_ to bind to decorin and biglycan helps protect growth factors from proteolytic degradation within the extracellular environment (26–30). In addition, there appears to be a role for these SLRPs in regulating TGF-β_1_ cell signaling activity. By binding to TGF-β_1_, decorin blocks the interaction with TGF-βRI/II receptors to prevent signaling via Smad2/3 and the ERK1/2 pathway, thereby preventing the over-activation of TGF-β_1_ and subsequent collagen overproduction and cell proliferation (28). Additionally, within mesangial cells, the binding of decorin to the epidermal growth factor (EGF) receptor has been shown to raise intracellular Ca^2+^ levels, which disrupt TGF-β_1_/Smad2 signaling (28).

Considering first the effect of long-term culture of CB-MSCs in normoglycemic conditions, our results indicated that intracellular, extracellular, and culture medium levels of TGF-β_1_ were largely unaffected during *in vitro* expansion. However, higher levels of synthesis and secretion of decorin were observed by CB-MSCs at PD150 compared to PD15. This would suggest that the two cell populations at PD15 and PD150 vary with respect to decorin, but not TGF-β_1_ biosynthesis. For CB-MSC cultures expanded to PD150 under hyperglycemic conditions, levels of TGF-β_1_ were dramatically reduced, which was consistent for TGF-β_1_-LAP homodimers, pre-pro-TGF-β_1_ monomers, TGF-β_1_ precursor proteins, and active TGF-β_1_ dimers. Levels of decorin synthesis and secretion were increased further when expanded in a high glucose culture environment, suggesting that glucose has differential long-term effects on the CB-MSCs. When assessing the short-term glucose exposure of CB-MSCs at PD15 and PD150, our collective results suggest that high glucose may have a greater influence on TGF-β_1_ and decorin synthesis of the more committed osteoblast cells present within the PD15 population, greatly increasing their secretion by these cells, which was not confidently observed for the transit-amplifying cells predominant in the PD150 population. It should be noted that less discernible or small subtle differences were observed when analyzing the effects of high glucose on biglycan synthesis and secretion.

Such conclusions are noteworthy, as the consensus of data suggests that TGF-β_1_ generally acts to inhibit mineralized matrix formation by committed osteoblasts (19), which may provide one mechanistic explanation for how high glucose inhibits bone-repair processes. If these observations translate to the *in vivo* situation, then severe reductions in TGF-β_1_ or an increase in decorin synthesis and secretion would be expected to have detrimental consequences for the amplification of the progenitor MSC population and the subsequent deposition of a collagen-rich ECM, thereby significantly disrupting normal bone (19).

An alternative source of TGF-β_1_ within the wound-healing environment is the M2 macrophage population (5, 6). Macrophages are now established to exist within a bone-healing site as one of two distinct phenotypes of M1 and M2 (5, 6). Our previous *in vivo* studies identified elevated macrophage levels associated with implant osseointegration in diabetic GK rats when compared to non-diabetic Wistar rats (12). However, as the precise phenotypic profiles of those macrophages predominant in diabetic bone tissues remains tentative with respect to the formation of M2, monocytes were isolated from the bone marrow of Wistar rat long bones and appropriately stimulated to promote the formation of the M1 or M2 phenotype. Within this study, our approach was to investigate, through *in vitro* studies, whether a hyperglycemic environment could affect M1 and M2 polarization and thus potentially influence the bone-healing process. Short-term exposure to high glucose levels was observed to hinder considerably the development of the M2 phenotype *in vitro*, evident by the significant reductions in M2 macrophage marker gene expression for TGF-β_1_, Arg1, VEGF, and CD163. Evidence suggests that for proper bone repair, the healing site is initially dominated by the pro-inflammatory M1 phenotype, where they are proposed to provide roles in phagocytosing bacteria and the apoptosis of neutrophils (15). Performing the latter function is further proposed to stimulate a prerequisite transition of macrophages to develop the M2 phenotype, which is critical in resolving inflammation, promoting angiogenesis, and early matrix deposition (5, 6, 15). Indeed, several studies have indicated that a failure in switching from an M1 to an M2 phenotype delays bone healing (45–47), and the high and prolonged presence of M1 macrophages generating high levels of pro-inflammatory cytokines, such as TNF-α, contribute to the pathogenesis of impaired wound healing associated with type 2 diabetes (12). Within the present study, we also noted significant reductions in IL-6 and iNOS, but not in TNF-α expression, upon monocyte differentiation to the M1 phenotype after high glucose exposure. This may indicate that macrophages are still capable of generating some form of a M1 macrophage-rich, pro-inflammatory environment, where TNF-α is a prominent mediator (11, 12, 45–47). It should be noted that IL-6 is described as a pleiotropic mediator, assigned roles in both mediating inflammation and bone tissue destruction, in addition to activities that aid the resolution of inflammation dependent upon its signaling route (55). Following a switch from a *classic* to a *trans* IL-6 signaling route, IL-6 is proposed to induce neutrophil apoptosis (55) and is implicated in the shift from pro-inflammatory M1 to anti-inflammatory M2 macrophages and in the promotion of MSC recruitment from their niches (56, 57). As overall inhibition in IL-6 during the repair phase has been demonstrated to disturb and delay fracture healing (56, 57), the observed reduction of IL-6 in high glucose conditions could, therefore, also contribute to the delay in bone healing associated with T2DM.

To conclude, this study has provided new information to indicate that *in vitro* exposure of high glucose levels on CB-MSCs can influence the synthesis and secretion of TGF-β_1_ and their regulatory SLRPs, depending upon the length of exposure. Moreover, our *in vitro* observations, where the short-term glucose exposure of cells at PD15 is associated with an increase in both TGF-β_1_ and decorin, correlate with our *in vivo* investigation presented herein, which demonstrated that TGF-β_1_ levels are higher in the healing collagenous matrix of young diabetic rats. Conversely, the downregulation of these vital matrix and signaling proteins, apparent after CB-MSC exposure to chronically high glucose concentrations, may be a contributory factor in the severe impairment to bone repair in aged, diabetic animals. In considering the role of TGF-β_1_ during the early stages of osteoblast differentiation, both increased and decreased levels of the growth factor and its matrix ligand binding partners of decorin and biglycan have the potential to delay the formation of a mature osteoblast and hence bone deposition (see Graphical Abstract). In drawing these conclusions, it is recognized that other influential factors, such as prolonged hyperinsulinemia and organismal aging reducing the proliferative and regenerative capacity of the mesenchymal progenitor cell populations (58), cannot be discounted in further contributing to the delayed bone healing associated with T2DM. Within this study, we additionally present evidence to suggest that bone healing may also be abrogated due to the delay in the formation of the M2 phenotype, which is compounded by known effects of high glucose levels in increasing macrophage populations (12). Interestingly, SLRPs, including decorin, have been shown to bind to the toll-like co-receptor CD14, where they have been proposed roles in stimulating inflammatory cell responses in macrophage activity (28, 36), and to CD44, to trigger autophagy and promote resolution of inflammation (36). This provides a significant paradigm shift, with implications for considering the regulation of inflammation in chronic systemic diseases, such as T2DM. Collectively, in recognizing the ever-increasing incidence of T2DM worldwide, the data presented contributes to an advancement of knowledge and understanding toward elucidating the pathogenic influence of hyperglycemia on bone healing, which may be of value in facilitating better clinical and pharmacological management for the disease in dental and orthopedic procedures.

## Data Availability

The original contributions presented in the study are included in the article/**Supplementary Material**, and further inquiries can be directed to the corresponding author.
