# Supplementary figures and images for: Hyperglycemia exerts disruptive effects on the secretion of TGF-β1 and its matrix ligands, decorin and biglycan, by mesenchymal sub-populations and macrophages during bone repair
Source: Front Dent Med. 2023 Jun 26;4:1200122. doi: 10.3389/fdmed.2023.1200122 (PMC11797960; doi:10.3389/fdmed.2023.1200122)

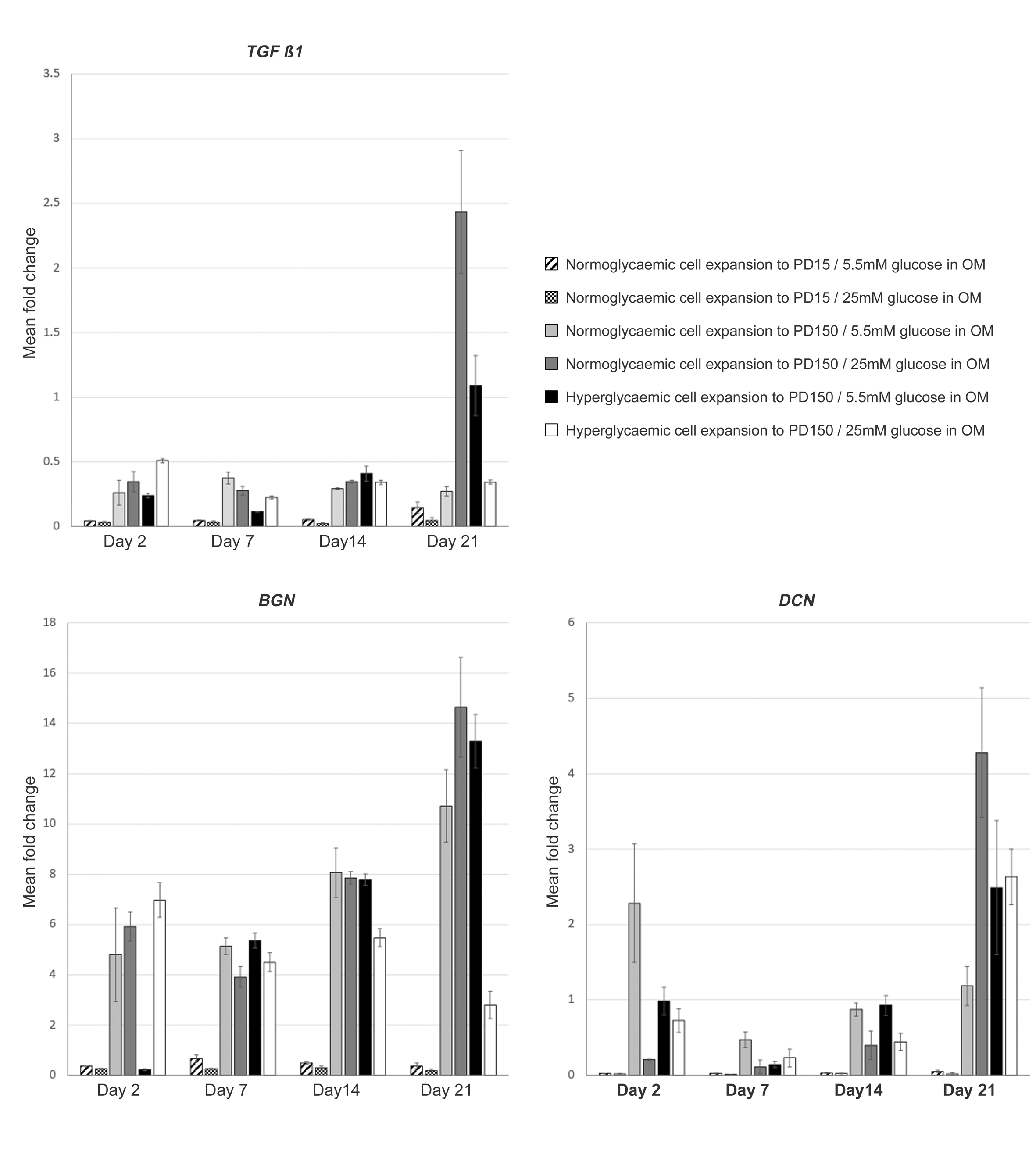

Supplement: Supplementary file 1 [file Image1.tif]
